# Porcine Follicular Fluid-Derived Exosome: The Pivotal Material for Porcine Oocyte Maturation in Lipid Antioxidant Activity

**DOI:** 10.3390/ijms24129807

**Published:** 2023-06-06

**Authors:** Euihyun Kim, Kihae Ra, Myung-Shin Lee, Geon A. Kim

**Affiliations:** 1 Department of Theriogenology and Biotechnology, College of Veterinary Medicine, Seoul National University, Seoul 08826, Republic of Korea; 2 Department of Microbiology and Immunology, School of Medicine, Eulji University, Daejeon 35233, Republic of Korea; 3 Department of Biomedical Laboratory Science, School of Healthcare Science, Eulji University, Uijeongbu 34824, Republic of Korea

**Keywords:** exosomes, follicular fluid, porcine, oocyte maturation, antioxidant activity, lipid metabolism

## Abstract

Several studies have examined exosomes derived from porcine follicular fluid (FF), but few have reported their application in controlled experiments. The main concern in the field of embryology may be that controlled conditions, such as using a defined medium intermittently, cause poor results in mammalian oocyte maturation and embryo development. The first reason is the absence of the FF, which copes with the majority of the processes emerging in oocytes and embryos. Therefore, we added exosomes derived from porcine FF to the maturation medium of porcine oocytes. For morphological assessment, cumulus cell expansion and subsequent embryonic development were evaluated. Moreover, several stainings, such as glutathione (GSH) and reactive oxygen species (ROS), fatty acid, ATP, and mitochondrial activity, as well as evaluations of gene expression and protein analysis, were used for the functional verification of exosomes. When the oocytes were treated with exosomes, the lipid metabolism and cell survival of the oocytes were fully recovered, as well as morphological evaluations compared to the porcine FF-excluded defined medium. Therefore, controlled experiments may produce reliable data if the exosomes are treated with the desired amounts, and we suggest applying FF-derived exosomes to promote experimental data when performing controlled experiments in embryology.

## 1. Introduction

Exosomes are a type of extracellular vesicle (EV) that measure approximately 30–200 nm in size [1]. An endosome, also known as a multivesicular body, is created in the endosomal lumen of cells and contains numerous types of intraluminal vesicles. The multivesicular body can either fuse with lysosomes for degradation or with the cell surface, and the intraluminal vesicles are secreted as exosomes [2,3,4]. It has consistently been reported that exosomes play several critical roles in mammalian reproduction. It has been suggested that FF-derived exosomes play fundamental roles in bovine oocyte maturation and subsequent embryonic development [5]. In addition, Gabrys et al. suggested that FF-derived exosomes are important modulators of nuclear maturation in mares [6]. In addition, Hung et al. reported that FF-derived exosomes significantly affect the expansion of cumulus cells (CCs), indicating that exosomes play a crucial role in reproduction [6,7]. Recently, it has been shown that exosomes derived from the mammalian reproductive system are crucial for gametes and embryos and that their functions depend on various sites in the reproductive system [2]. Therefore, exosomes have emerged as essential factors in mammalian reproduction.

FF has been widely studied and is known to have pivotal functions, such as the maturation of mammalian oocytes, control of reproductive hormones, and regulation of proteins [8,9,10]. Therefore, the use of FF in mammalian reproduction is necessary. However, numerous studies in the field of mammalian embryology have excluded FF from in vitro oocyte maturation or embryo development media (so-called defined media) for controlled experiments, as it is first collected from different ovaries and has minor differences in concentration or compounds every time it is collected. Thus, most of the results using the defined medium indicated the poor maturation of oocytes or the development of embryos in the control groups. This implies that the absence of FF critically affects the metabolism of mammalian oocytes and embryos. Although research has shown the improved effectiveness of the selected chemicals or experimental designs, poor results in the control groups still require better standardization.

The causes of these poor results may be its role at the molecular level. It has been reported that fatty acids in porcine FF (pFF) affected the lipid metabolism of cumulus–oocyte complexes (COCs) [11]. Moreover, Pawlak et al. suggested that the competence of porcine oocytes and embryos depends on the fatty acid profiles of FF collected from prepubertal and cyclic gilts [12]. In addition, it is reported that pFF influences cell proliferation, steroid synthesis [13,14,15], and MAPK/ERK and WNT/B-Catenin signaling pathways [16]. Several studies have linked factors that affect metabolism to EVs derived from FF or EVs themselves, which contain maturation-related proteins, mRNA, lipids, and miRNA that are closely associated with lipid metabolism [6,17,18]. Therefore, we compared pFF and pFF-derived EVs in porcine oocyte maturation to determine whether the EVs can substitute the roles of pFF, which enables its use in controlled experiments by adjusting the concentration or number of EVs. In addition, we aimed to determine the effects of pFF-derived EVs on lipid biogenesis and mitochondrial function during porcine oocyte maturation.

## 2. Results

### 2.1. Characterization of PFF-Derived EVs and EV Treatments on Porcine Oocytes

**Figure 1:**
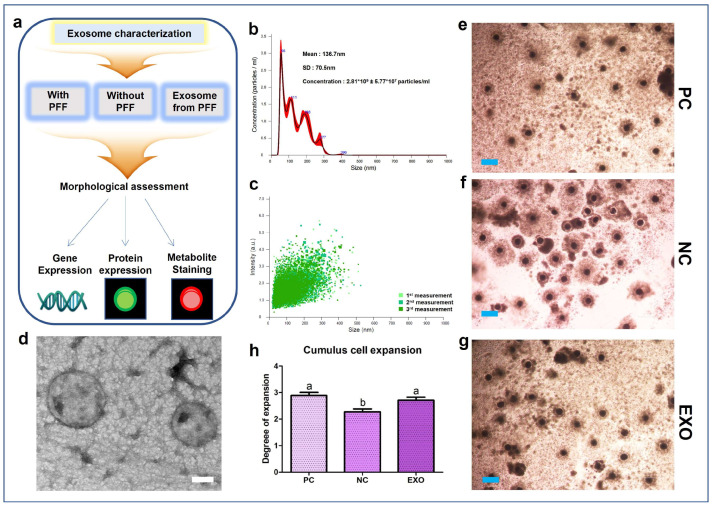
Characterization of pFF-derived exosomes and assessment of CC expansion of porcine COCs. (**a**) Summarized experiment flow of the study. The size of the pFF-derived exosome was evaluated by NTA (**b**,**c**) and morphology of the exosomes was observed through TEM (White bar: 50 nm) (**d**). Assessment of the CC expansion of porcine COCs was performed by microscopy-based images of the COCs (Blue bar: 200 μm) (**e**–**g**), and the CC expansion rates were calculated with statistics (**h**). For the assessment of CC expansion, four biological replications were performed and more than 210 oocytes from each experimental group were used. Data are shown as the means ± S.D. Groups marked with different alphabetical letters are significantly different (*p* < 0.05). PC, positive control; NC, negative control; EXO, pFF-derived exosome treated group.

The EVs were extracted from 1 mL of pFF in a fixed volume for the in vitro maturation (IVM) of porcine oocytes, and the same volume was subsequently added to the defined medium for an exact comparison of the experiment. The pFF-derived EVs were first characterized by Nanoparticles Tracking Analysis (NTA), and we found that most EVs were detected at the size of 50–200 nm, with an average of 136.7 nm (Figure 1b,c). Moreover, it was shown that the predicted number of EVs was 2.81 × 10^9^ particles/mL, which was a 10-fold dilution of 1 mL EVs. Hence, 2.81 × 10^10^ particles/mL was added to the IVM medium for oocyte maturation. In addition, the EVs were characterized by transmission electron microscopy (TEM), which showed that the size of the vesicles was within an acceptable range (Figure 1d). The expansion of CCs was evaluated after treatment with EVs. The EV-treated groups showed no significant differences compared to the positive control (PC) group. In contrast, the negative control (NC) group, which was not treated with pFF or EVs, showed a significant decrease among the groups (Figure 1e–h). This implies that culturing oocytes in a defined medium may result in insufficient maturation.

### 2.2. GSH/ROS Measurement

**Figure 2:**
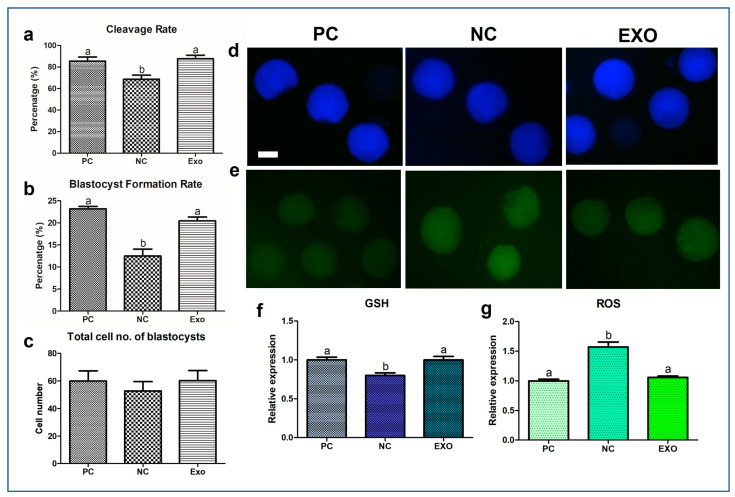
Evaluation of subsequent embryonic development and assessment of ROS and GSH staining of porcine oocytes. Cleavage rate (**a**), blastocyst formation rate (**b**), and total cell number of blastocysts (**c**) were monitored and evaluated for 7 days. The porcine oocytes were stained with GSH and ROS, then their fluorescence intensities were analyzed (**d**–**g**). More than 190 embryos in each experimental group from six biological replications were used and more than 30 oocytes from three biological replications were used for GSH and ROS staining. Bars with different alphabetical letters are significantly different among the groups (*p* < 0.05). White bar: 50 μm. PC, positive control; NC, negative control; EXO, pFF-derived exosome treated group.

After the maturation and activation of the oocytes, development was monitored for 7 d. As the result, the NC group showed a significant decrease in cleavage rate and blastocyst formation rate compared to those in the PC- and exosome-treated groups (cleavage rate: 68.66% vs. 85.47% and 87.74% *p* < 0.05; blastocyst formation rate: 12.49% vs. 23.16% and 20.45%, respectively, *p* < 0.005) (Figure 2a,b). These results indicate that the exosome-treated (EXO) group has a developmental potential similar to that of the PC group. However, none of the groups showed significant differences in the total blastocyst cell number (Figure 2c). Subsequently, we measured the levels of GSH and ROS in each group. The results (Figure 2d–g) were somewhat distinct, in that the NC showed a significant decrease in GSH expression levels (*p* < 0.05) and an increase in ROS expression levels (*p* < 0.005), implying that the EVs firmly intervened in the antioxidant system in porcine oocytes.

### 2.3. MRNA Gene Expression in Porcine Oocytes

**Figure 3:**
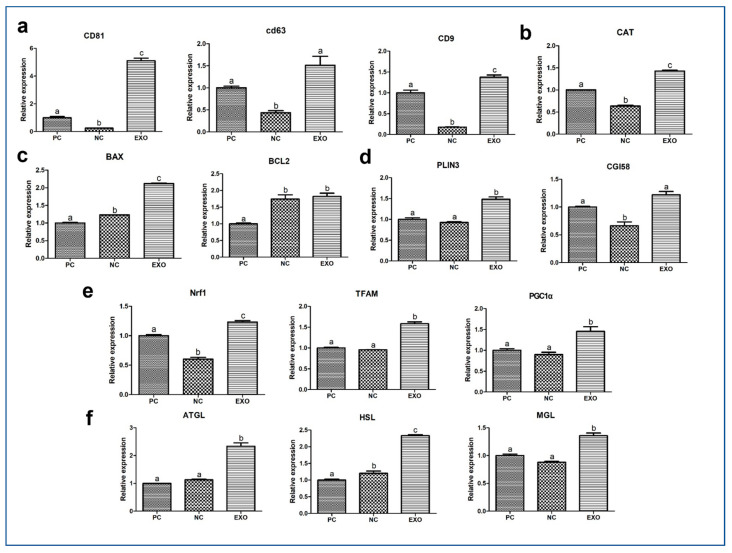
Relative quantitative mRNA expression through real-time PCR. The relative expression of mRNA transcript related to exosome markers (*CD81*, *CD63*, and *CD9*) (**a**), antioxidant activity (*CAT*) (**b**), apoptosis (*BAX* and *BCL2*) (**c**), lipid storage and breakdown (*PLIN3* and *CGI58*, respectively) (**d**), mitochondria (*Nrf1*, *TFAM*, and *PGC1α*) (**e**), and lipolysis (*AGTL*, *HSL*, and *MGL*) (**f**) are shown for the three different experimental groups: PC, positive control; NC, negative control; EXO, pFF-derived exosome-treated group. The experiments were performed in triplicate and at least 290 oocytes from each experimental group used for the analysis were collected from seven biological replications. Bars with different alphabetical letters are significantly different among the groups (*p* < 0.05).

First, the mRNA expression of the exosome markers cluster of differentiation (CD) 81, CD63, and CD9 showed extensive differences among the groups (Figure 3a). All genes showed significant differences in mRNA expression between the PC and NC groups, and the EXO group showed a significant increase compared to the NC group (*p* < 0.05). The expression of *catalase* (*CAT*) also showed a significant increase in the EXO group compared to that in the PC and NC groups (Figure 3b; *p* < 0.05). The mRNA expression of *Bcl-2-associated X protein* (*BAX*) and *B-cell lymphoma 2* (*BCL2*) was the lowest in the PC group (Figure 3c, *p* < 0.05). The expression of *Perilipin3* (*PLIN3*) was higher in the EXO group than in the other groups (Figure 3d, *p* < 0.05). The expression levels of *nuclear factor erythroid 2-related factor 1* (*Nrf1*), *mitochondrial transcription factor A* (*TFAM*), and *Peroxisome proliferator-activated receptor-gamma coactivator-1alpha* (*PGC1α*), mitochondria-related genes, showed a higher increase in the EXO group than in the NC and PC groups (Figure 3e, *p* < 0.05). In addition, *adipose triglyceride lipase* (*ATGL*), *hormone-sensitive lipase* (*HSL*), *monoacylglycerol Lipase* (*MGL*), and *comparative gene identification-58* (*CGI-58*), lipolysis-related genes, showed higher expression levels in the EXO group than in the PC and NC groups (Figure 3f, *p* < 0.05).

### 2.4. Patterns of Protein Expression Evaluated by ICC, BODIPY, and JC-1 Matrix Metalloproteinase (MMP) Staining

**Figure 4:**
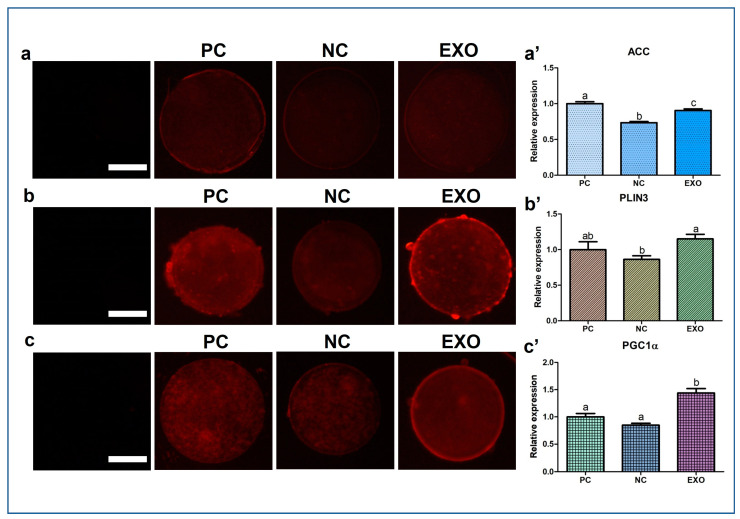
Immunocytochemical analysis of ACC, PLIN3, and PGC1α in porcine oocytes and fluorescence microscopy was applied to obtain images of the oocytes (**a**–**c**). Representative oocytes in each group were stained with ACC, PLIN3, and PGC1α rabbit-derived antibody and analysis of intensities from ACC-, PLIN3-, and PGC1α-stained oocytes (**a**′–**c**′). At least 19 oocytes from four biological replications in each group were used for the staining and immunocytochemistry was performed three times technically. Data are shown as the means ± S.D. Bars with different alphabetical letters are significantly different (*p* < 0.05). PC, positive control; NC, negative control; EXO, pFF-derived exosome treated group. White bars in the images indicate 50 µm; 400× magnification.

**Figure 5:**
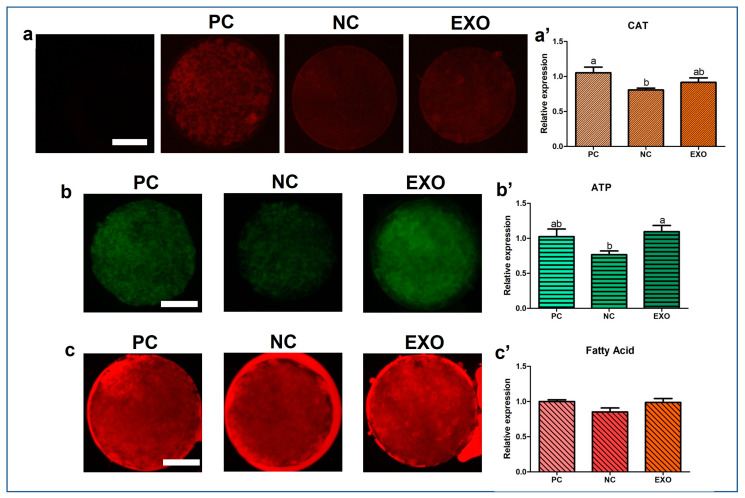
Immunocytochemical analysis of CAT and BODIPY staining analysis of ATP contents and fatty acids in porcine oocytes. Fluorescence microscopy was applied to obtain images of the stained oocytes. Representative oocytes in each group were stained with CAT rabbit-derived antibody (**a**), BODIPY FL ATP and BODIPY 558/568 C12, respectively (**b**,**c**). Analysis of intensities from CAT antibody-stained oocytes (**a**′) and BODIPY FL ATP- and BODIPY 558/568 C12-stained oocytes (**b**′,**c**′). At least 21 oocytes from four biological replications (Immunocytochemistry of CAT) and five biological replications (BODIPY staining) in each group were used for the staining and it was performed three times technically. Data are shown as the means ± S.D. Bars with different alphabetical letters are significantly different (*p* < 0.05). PC, positive control; NC, negative control; EXO, pFF-derived exosome treated group. White bars in the images indicate 50 µm; 400× magnification.

**Figure 6:**
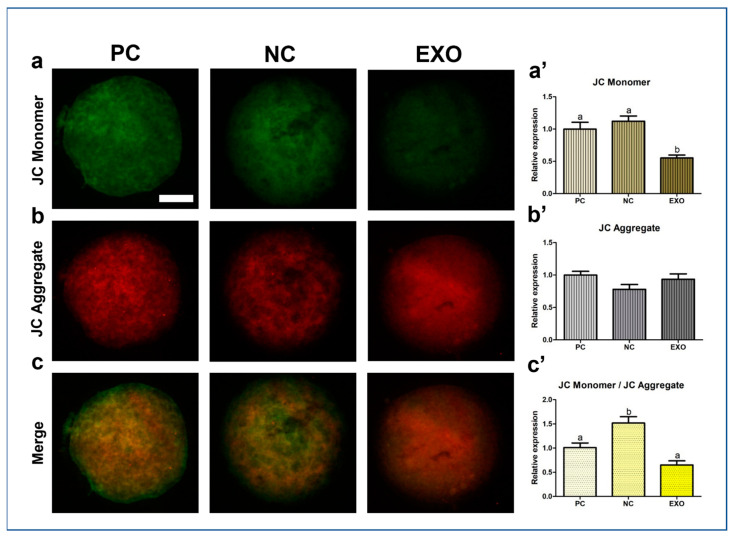
JC-1 mitochondrial membrane potential (MMP) staining in porcine oocytes. Fluorescence microscopy was applied to obtain images of the oocytes. Representative blastocysts in each group were stained JC-1 monomer (**a**) and JC-1 aggregate (**b**), then the images were merged for the calculation of ratio of changes (**c**). Analysis of intensities from JC-1 MMP-stained oocytes (**a**′–**c**′). At least 20 oocytes from four biological replications in each group were used for the staining and it was performed three times technically. Data are shown as the means ± S.D. Bars with different alphabetical letters are significantly different (*p* < 0.05). PC, positive control; NC, negative control; EXO, pFF-derived exosome treated group. White bars in the images indicate 50 µm; 400× magnification.

Figure 4 and Figure 5a show the expression patterns of Acetyl-CoA carboxylase (ACC), PLIN3, PGC1α, and CAT. In ACC protein expression the exosome-treated group was significantly higher than that in the NC group (Figure 4a,a′, *p* < 0.005), but not as much as that in the PC group, and its expression level in PLIN3 was also higher than that in the NC group (Figure 4b,b′, *p* < 0.05); however, no difference was observed between the exosome-treated group and the PC group. For PGC1α, the protein expression was higher in the EXO group than in the other control groups (Figure 4c,c′, *p* < 0.05). In addition, the exosome-treated group did not restore the expression of CAT as much as the PC group, with no differences among the control groups (Figure 5a,a′, *p* < 0.05). ATP and fatty acid contents were measured by BODIPY staining and subsequently analyzed. The EXO group showed significantly higher ATP recovery than the NC group (Figure 5b,b′, *p* < 0.05), and no differences were observed in fatty acids (Figure 5c,c′). Finally, the JC monomer was lower in the EXO group than in the other control groups (Figure 6a,a′, *p* < 0.05), whereas no differences were observed in JC aggregates (Figure 6b,b′). Most importantly, the ratio of JC monomers to JC aggregates was the highest in the NC group, indicating that the MMP intensity fluctuation at each wavelength was critically changed (Figure 6c,c′, *p* < 0.05).

## 3. Discussion

Researchers have been hesitant to address the challenge of achieving successful maturation of oocytes and development of embryos in mammalian gametes when using defined media in experiments; mammalian gametes are known to be sensitive and prone to poor maturation of oocytes or development of embryos when using defined media in experiments. Hence, we propose a simple yet fundamental experiment to improve mammalian oocyte maturation using a defined medium with a controlled number of EVs, as this issue needs to be addressed, sooner or later. Our results suggest that controlling the concentration of EVs in defined media, such as combinations of TCM199, hormones, and inorganic and organic substances, could partially restore the function of maturation media supplemented with FF by intervening in lipid metabolism and its subsequent downstream processes. The simplified experimental flow of our study design (Figure 1a) supports the field of mammalian reproduction by providing a framework for controlled experiments, such as testing the efficacy of unknown chemicals on mammalian oocyte maturation without FF or serum. However, numerous studies have been published regarding the key factors of pFF [16,19,20,21] and tests for unknown chemicals during mammalian oocyte maturation or embryo development with or without defined media [22,23,24,25]. Although studies using defined media may be suitable for testing unknown chemicals, many have shown poor oocyte development. Therefore, it is necessary to improve the oocyte quality while maintaining controlled conditions. Moreover, it is necessary to study, at least in part, why and how oocytes or embryos with insufficient development or maturation are obtained. Therefore, we chose the lipid metabolism–energy production pathway, which is one of the fundamental mechanisms of oocyte maturation [26,27,28,29].

Primarily, exosomes have already been studied in a variety of fields, demonstrating that vesicles contain diverse factors, such as proteins, RNAs, DNAs, and antibodies [30,31,32,33], and their presence in pFF has also been reported in several studies [2,16,34]. We believe that these studies provide sufficient evidence to support the idea that exosomes can be a substitute for pFF. Based on the assessment of CC expansion, the EV-treated group showed recovery, including subsequent embryonic development (Figure 1 and Figure 2). From our results (Figure 2d–g), the exosomes derived from pFF have an antioxidant ability, which corresponds to the study by Yuan et al., suggesting that exosomes derived from porcine theca cells increased antioxidation, proliferation, and steroid hormone synthesis [35]. Moreover, Zhang et al. reported that intracellular ROS generation leads to an increase in the number of multivesicular bodies, thereby releasing more exosomes through the synergistic effect of exofacial depletion of GSH [36]. CD81, CD63, and CD9, members of the tetraspanin family, are known exosome markers of the endosomal pathway [37]. Figure 3a shows the mRNA expression of these markers. We observed that the NC group showed distinctively decreased expression levels of these markers; however, the expression of these markers in the EXO group significantly increased by many folds. This may be explained by the activation of exosomes during isolation. Xu et al. suggested that the isolation process may cause changes in the size, shape, and protein contents of exosomes, affecting their functions and applications in clinical trials [38]; additionally, a study by Lobb et al. showed that the functions of exosomes from human plasma and cell culture supernatant were regulated depending on the isolation methods [39]. This can be interpreted as isolated exosomes having different properties and better functions than intact exosomes.

We observed a significant recovery in antioxidant properties, the results of which are shown in Figure 3b and Figure 5a,a′, indicating that exosomes mediate oxidative stress, as does pFF, which is supported by a previous study by Saeed-Zidane et al., in which the antioxidant properties were found to be regulated by exosomes in bovine granulosa cells [40]. In addition, genes and proteins related to lipid metabolism [41], *PLIN3,* and *CGI58* (Figure 3d), including the data from Figure 4b,b′, were also significantly recovered by pFF-derived exosomes. These results indicate that pFF and its derivatives closely mediate lipid metabolism, which carries fatty acids and their related factors [6,17,18,42]. The associated mechanisms with these genes can be linked to the results of our previous study conducted via transcriptome sequencing, thereby focusing on the impact of lipid metabolism on the competence of porcine oocytes and embryos [43,44,45]. We propose that lipid metabolism is significantly regulated by peroxisomal and mitochondrial activity, mediating antioxidant mechanisms through the Nrf2/ARE signaling pathway [46] and ATP production. Likewise, similar patterns (Figure 3e,f, Figure 4 and Figure 5a,a′) were observed in this study, showing the significance of lipid metabolism in porcine oocytes. This suggests that the NC group showed negative effects when pFF or exosomes were not treated, which could be a possible cause of poor competency in oocyte maturation and subsequent embryonic development in controlled experiments. As shown in Figure 5 and Figure 6, lipid metabolism and its subsequent products, which contribute to energy and antioxidant properties, are known to be the main factors responsible for its activity [47,48].

ACC plays a crucial role in catalyzing the carboxylation of acetyl-CoA to form malonyl-CoA, which is a fundamental precursor in the synthesis of fatty acids [49]. ACC also stores fatty acids and energy by inhibiting the transportation of triglycerides to the mitochondria [50]. We assume that the fatty acids (Figure 5c,c′) with no differences among the groups might partially be explained by the fact that the functions of ACC and lipolysis groups (*AGTL*, *HSL*, and *MGL*) are opposite. The main functions of lipolysis are the breakdown of fatty acids and the transportation of short-chain fatty acids to the mitochondria for subsequent energy production [51,52]. Our results show that both ACC (fatty acid storage) and *ATGL*, *HSL*, and *MGL* (fatty acid breakdown) were upregulated in the EXO group. Therefore, we speculate that both pathways have a positive and negative feedback. Significant differences from the given data were shown in ATP, but not in fatty acids; however, the membrane potential of mitochondria in porcine oocytes was significantly influenced when only pFF-derived exosomes were used. As we have mentioned the roles of lipid synthesis and lipolysis, we expect that the reason why mitochondria were affected by exosomes may be due to the lipolysis of fatty acids (partially originating from exosomes) [53].

## 4. Materials and Methods

### 4.1. Chemicals and Research Ethics

Most of the reagents and chemicals used in this study were obtained from Sigma–Aldrich Chemical Company otherwise indicated. Porcine ovaries were provided from a local slaughterhouse, after manufacturing processes. The use of porcine ovaries in this study was approved by the Institutional Animal Care and Use Committee (IACUC) of Seoul National University (Approval ID: SNU-190621-2-1).

### 4.2. Exosome Isolation and Characterization

The pFF was collected from porcine ovarian follicles and aspirated with sterilized 10-gauge needle-syringe. Immediately after the collection of the pFF, it was centrifuged at 2000× *g*, 4 °C, 30 min to remove cells and debris. Then, the supernatant was transferred to a new tube without disturbing pellets and centrifuged again at 10,000× *g*; 4 °C for 30 min. Subsequent procedures were followed by the manufacturer’s protocol. Briefly, the centrifuged supernatant was mixed with Total Exosome Isolation reagent (4484453, Invitrogen, Vilnius, Lithuania) then, incubated at room temperature for 30 min. After the incubation, the mixture was centrifuged at 10,000× *g*, 2 °C for 1 h. The supernatant was carefully removed then; the pellet was resuspended with PBS. Isolated exosomes were kept at −80 °C for less than a week and used for further analysis and experiments. The characterization was followed by the study of Sadelddin et al. [54]. Briefly, the isolated EVs were firstly characterized by NTA (LM10, Nanosight, Malvern PANalytical, UK) for determining the size and concentration of the EVs. Secondly, TEM was used for examining the EVs. The suspended pellets were mounted on 300-mesh grids, then dried and stained with 2% uranyl acetate. The EVs were visualized by an energy-filtering TEM (LIBRA 120, Jena, Germany) at 120 kV.

### 4.3. Retrieval of COCs and IVM

The entire process of IVM was described previously [55]. Briefly, the ovaries were delivered from local abattoir at 38 °C saline. After the process of washing with the saline, retrieval of COCs was performed by aspirating follicles with sterilized 10-gauge needle-syringe, then washed three times in washing medium. COCs with homogenous cytoplasm, distinct cellular membrane, and more than three layers of CCs were selected, then they were cultured in IVM medium. The immature COCs were firstly cultured with hormones (human chorionic gonadotropin and equine chorionic gonadotropin) for 20–22 h, and then cultured without the hormones for additional 20–22 h at 39 °C under 5% CO2 in 95% humidified air. Experimental groups are as follows: Positive control (PC); maturation medium containing pFF, Negative control (NC); maturation medium without pFF, and experimental group; maturation medium containing pFF-derived exosome. In case of the EV treatment during porcine oocyte maturation, approximately 2.81 × 10^10^ particles/mL were treated as the extracted EVs were diluted 10 times for the evaluation result from NTA.

### 4.4. Cumulus Cell Expansion Assessment

The evaluation of CC expansion was performed after 42–44 h of IVM. The entire procedure of the CC expansion is described in detail in our previous works [43]. Briefly, the expansion rate was processed in the following manner: Degree 4; full expansion of the CCs including the layer of corona radiata, which is positioned the closest to the oocyte. Degree 3; expansion of all layers of the CCs except for the corona radiata. Degree 2; expansion at only the outermost layer of the CCs. Degree 1; the CCs are in a spherical shape and observed only a single layer. Degree 0; the CCs are present as flattened monolayer with fibroblastic appearance, being complete detached from the oocyte.

### 4.5. Parthenogenetic Activation (PA)

The entire process of PA was previously described [44]. The COCs are denuded with 0.1% hyaluronidase after 44 h of IVM. Then, the denuded oocytes were washed several times and selected in Tyrode’s albumin lactate pyruvate (TALP) medium. Oocytes that have first polar body with homogenous cytoplasm and distinct cellular membranes were gently equilibrated in activation medium (serially diluted with TALP) containing 0.1 mM MgSO4, 0.28 M mannitol, 0.1 mM CaCl2 and 0.5 mM HEPES. The equilibrated oocytes were then transferred to the activation medium in a 3.2 mm double electrode chamber. Activation was performed by a direct current pulse of 1.5 kV/cm with 60 μs electric stimulation using a BTX Electro-Cell Manipulator 2001 (BTX Inc., San Diego, CA, USA). The electrically activated oocytes were thereafter washed and re-stabilized in porcine zygote Medium-5 (PZM-5; CSR-CK024; Waco Chemicals, Osaka, Japan). Lastly, they were transferred to 30 μL droplets of PZM-5, covered with mineral oil and cultured at 39 °C in a humidified atmosphere of 5% CO2, 5% O2, and 90% N2 for 7 days.

### 4.6. Embryo Evaluation and Total Cell Count after PA

The day on which the electrically activated oocytes were transferred to the in vitro culture (IVC) medium (PZM-5) was considered as Day 0. After 48 h of culture, counting of evenly cleaved embryos was performed under a stereomicroscope and dead, degenerated, or one-cell embryos were ruled out. On Day 7 (168 h of culture), the numbers of blastocysts were counted; then, they were washed in PBS for several times and fixed for 2 h in 4% paraformaldehyde (*w*/*v*) in PBS at room temperature. Subsequently, the blastocysts were stained with 5 μg/mL of Hoechst 33,342 for 10 min. Subsequently, the blastocysts were washed again with PBS to remove the stain, then mounted on glass slides and covered with cover slips. The total cell numbers of the blastocysts were counted under a fluorescence microscope (Nikon Corp., Tokyo, Japan) at 400× magnification.

### 4.7. Immunofluorescence Staining

Matured COCs were denuded with 0.1% hyaluronidase in TALP and then fixed with 4% paraformaldehyde (*w*/*v*) in PBS for 2 h at room temperature. Permeabilization of the oocytes was processed with 1% Triton X-100 (*v*/*v*) in distilled water (DW) for 1 h at 39 °C and washed four times in 1% PVA/PBS. Then, for blocking, the permeabilized oocytes were incubated in 2% BSA in 1% PVA/PBS for at 2 h to prevent non-specific bindings. Subsequently, the oocytes were directly transferred to 2% BSA containing primary antibody for ACC (1:200; A5-17564; Thermo Fisher Scientific, Waltham, MA, USA), as well as PLIN3 (1:200; ab47638; Abcam, Cambridge, UK), PGC1α (1:200; PA5-38021; Thermo Fisher Scientific, Waltham, MA, USA), and CAT (1:200; 21260-1-AP; Proteintech, Rosemont, IL, USA) and incubated overnight at 4 °C. After the incubation, the oocytes were then washed several times in 1% PVA/PBS and incubated with a secondary fluorescein isothiocyanate-conjugated Goat Anti-Rabbit IgG H&L (Texas Red^®^, 1:200; ab6719; Abcam, Cambridge, UK) at 37 °C for 2 h (light avoided). After the secondary antibody incubation and washing with 1% PVA/PBS, the counterstaining of the oocytes was immediately performed with 5 μg/mL Hoechst-33342 for 10 min. After thorough washing, they were mounted on glass slides with 100% glycerol droplets, covered with cover slips, and then analyzed under a fluorescence microscope. The fluorescence was evaluated while using ImageJ software (version 1.46r; National Institute of Health, Bethesda, MD, USA). Within three independent replications, at least 20 oocytes from each group were used for the staining.

### 4.8. ATP Content Assay

Matured COCs were denuded and then fixed in 4% PFA/PBS for 2 h at room temperature. Subsequently, the fixed oocytes were washed in 1% PVA/PBS droplets for three times, and they were transferred to 0.5 μM of BODIPY FL ATP (BODIPY-ATP; A12410; Molecular Probes, Eugene, OR, USA) in PBS for 1 h at room temperature in the dark. After the staining, the oocytes were again washed in PBS and mounted on glass slides and covered with slips. An epifluorescence microscope (TE2000-S; Nikon, Tokyo, Japan) was used for capturing images, and the intensities of the ATP contents were measured while using ImageJ software (version 1.46r; National Institutes of Health, Bethesda, MD, USA). The intensities of the control group were standardized to 1. At least 20 oocytes from each experimental group were used for the staining.

### 4.9. Measurement of Intracellular GSH and ROS Levels

After 42–44 h of IVM, the matured COCs were denuded and washed several times thoroughly in TALP medium. For measuring the intracellular ROS and GSH levels in oocytes, H2DCFDA (2′, 7′-dichlorodihydrofluorescein diacetates; Invitrogen, Waltham, MA, USA) and CellTracker Blue (4-chloromethyl-6.8-difluoro-7-hydroxycoumarin; CMF2HC; Invitrogen) were used, respectively. The oocytes were then transferred to 10 μM of CellTracker Blue or 10 μM of H2DCFDA in TALP medium and incubated for 30 min at room temperature (Light avoided). Stained oocytes were washed several times in TALP medium, and then they were transferred to a 4 μL droplet of TALP medium and covered with mineral oil. Epifluorescence microscope (TE2000-S; Nikon, Tokyo, Japan) was used for measuring the intensities of the fluorescence, observed through UV filters (460 nm for ROS and 370 nm for GSH), then the images were captured. Analysis was performed by Image J software and the intensities of the control group was standardized to 1. For three independent replications, at least 36 oocytes from each experimental group were used in this experiment.

### 4.10. Fluorescent FA Analog Assays

The fluorescent FA analog assay was performed according to a study by Jin et al. [56]. In brief, the matured oocytes were denuded and washed fixed in 4% PFA/PBS for 1 h at room temperature. The fixed oocytes were then incubated in 6 M BODIPY 558/568 C12 (BODIPY-FA; D3835; Molecular Probes, Eugene, OR, USA) diluted in PBS for 1 h at room temperature, avoiding light. After the incubation, the stained oocytes were again washed three times in PVA/PBS before mounting on glass slides with cover slips. Images were captured using an epifluorescence microscope (TE2000-S; Nikon, Tokyo, Japan) and the fluorescence intensities of the FA were evaluated using ImageJ software. The intensities of the control group were standardized to 1. The staining was performed at least three times technically in each group and at least 18 oocytes from six biological replications were used for the staining.

### 4.11. JC-1 MMP Assays

The entire process was followed and modified by the manufacturer’s protocol. The matured porcine oocytes were fixed for 2 h at room temperature and washed in 1% PVA/PBS, then incubated at 37 °C in JC-1 solution mixed with 1% PVA/PBS for 30 min. After the incubation, the oocytes were washed several times, and then mounted on cover slips. Images of each oocyte were captured by an epifluorescence microscope (TE2000-s; Nikon, Tokyo, Japan). The fluorescence ratio of JC-1 monomer and aggregate (530 nm and 590 nm, respectively) was evaluated using ImageJ software (version 1.46r; National Institutes of Health, MD, USA). The intensity values of the control group were standardized as 1. The staining was conducted for three technical replications from 5 biological replications. At least 21 oocytes from each group were used for the staining.

### 4.12. Analysis of Gene Expression by Quantitative Real-Time PCR (qRT-PCR)

After denuding matured porcine oocytes, they were washed with PBS, then stored at −80 °C until RNA extraction. At least 389 oocytes from each experimental group were used for RNA extraction using the RNAqueous^TM^ Micro Kit (Invitrogen, Vilnius, Lithuania). The measurement of quantified mRNA was performed by a NanoDrop 2000 Spectrophotometer (Thermo Fisher Scientific, Wilmington, MA, USA), and then complementary DNA (cDNA) was synthesized by amfiRivert cDNA synthesis Platinum Master Mix 0 (genDEPOT, Houston, TX, USA) in accordance with the manufacturer’s protocol. For the performance of qRT-PCR, reaction mixtures containing 0.4 μL (10 pmol/μL) forward primer, 0.4 μL (10 pmol/mL) reverse primer, 8.2 μL of Nuclease Free Water (NFW), 10 μL SYBR Premix Ex Taq (Takara, Otsu, Japan), and 1 μL cDNA in a PCR plate (Micro-Amp Optical 96-Well Reaction Plate, Applied Biosystems, Singapore) were pre-mixed. Primer sequences are listed in Table 1. With the Applied Biosystems StepOnePlus^TM^ Real-Time PCR Systems (Applied Biosystems, Waltham, MA, USA), the amplification was performed with the following parameters: Forty cycles of reactions; denaturation for 15 s at 95 °C, annealing for 1 min at 60 °C and 1 min of extension at 72 °C. The oocytes were collected from at least three biological replications and three technical replications were performed in one plate. The expression of each target gene was quantified relative to that of the endogenous control gene (*GAPDH*). The calculation of the relative expressions was performed by the following equation:

R = 2 − [ΔCt sample − ΔCt control]

### 4.13. Statistical Analysis

At least three replications were performed in each experiment and for statistical analysis, GraphPad PRISM 5.01 (PRISM 5, GraphPad Software, Inc., San Diego, CA, USA) was used. For determining significant differences among the experimental groups, data were expressed as the mean ± S.D, analyzed using unpaired *t* test and univariate analysis variance (ANOVA) with Tukey’s Multiple Comparison Test. *p* values less than 0.05 were considered as significant difference among the experimental groups.

## 5. Conclusions

It is generally accepted that the quality of porcine oocytes is evaluated by their developmental competencies and their survival in the in vitro environment. Particularly, the antioxidant mechanism and energy production through fatty acid-related signaling pathways could be the main parameters for assessing the quality of porcine oocytes. Overall, when porcine oocytes were treated solely with pFF-derived exosomes, the function of pFF was completely restored, mimicking and regulating lipid metabolism and its subsequent cascades. Here, we concluded that pFF-derived exosomes are a perfect substitute for pFF because they recover and maintain the maturation levels of the oocytes. However, further studies on the contents of the exosomes derived from pFF need to be conducted to discover the exact functional match between pFF and pFF-derived exosomes.

## Figures and Tables

**Table 1:** Sequence-specific primers used for quantification of differentially expressed transcripts.

Genes	* Primer Sequences (5′–3′)	Product Size (Bp)	Accession No.
*GAPDH*	F: GTCGGTTGTGGATCTGACCT R: TTGACGAAGTGGTCGTTGAG	207	NM_001206359
*CATALASE*	F: AGGGAGAGGCGGTTTATTGC R: GGACTCGTTGGTGAAGCTCA	117	NM_001206359
*CD9*	F: CCGTGGTGATGATTTTCGGC R: ACAGGACCCCGAGAAGATGA	147	NM_214006.1
*CD63*	F: ACGTTCTTCTGCTGGCCTTT R: ACTGCGATGATGACCACAGG	136	XM_005663878.2
*CD81*	F: GGGTGCTGTGATGATGTTTG R: GCCTGGTCGTAGAACTGCTT	108	NM_001078679.1
*PGC1A*	F: CACGGACAGAACTGAGGGAC R: ACCTGCGCAAAGTGTATCCA	192	XM_021100442.1
*NRF1*	F: CAGCAAGTACAGCAGGTCCA R: ATGAGGCCGTTTCCGTTTCT	222	XM_021078993.1
*TFAM*	F: GCTCTCCGTTCAGTTTTGCG R: ACCTGCCAGTCTGCCCTATA	238	NM_001130211.1
*ATGL*	F: GACGGTGGCATCTCAGACAA R: TGGATGTTGGTGGAGCTGTC	113	NM_001098605.1
*HSL*	F: GCCTTTCCTGCAGACCATCT R: CACTGGTGAAGAGGGAGCTG	104	NM_214315.3
*MGL*	F: ACCCCACAGAGTGTCCCATA R: GGGTGTAGCTGAGGGTTTCC	96	XM_013982013.2
*CGI58*	F: TCTTGCTGGGACACAACCTG R: CCAAAGGGTCCTGCAATCCT	220	NM_001012407.1
*PLIN3*	F: ATGTTTGCCAGCGAGACAGA R: GTAGGCAGCAGACACCATGT	141	NM_001031778.1
*BAX*	F: CATGAAGACAGGGGCCCTTT R: CATCCTCTGCAGCTCCATGT	181	XM_003127290
*BCL2*	F: AATGTCTCAGAGCAACCGGG R: GGGGCCTCAGTTCTGTTCTC	193	NM_214285

* F, Forward primer; R, Reverse Primer.

## Data Availability

The data is contained within the article.

## Abbreviations

ACC	Acetyl-CoA carboxylase
ANOVA	Univariate analysis variance
ATGL	Adipose triglyceride lipase
BAX	BCL2-associated X protein
BCL2	B-cell lymphoma 2
CAT	Catalase
CC	Cumulus cells
CD	Cluster of differentiation
CGI58	Comparative gene identification-58
CMF2HC	4-chloromethyl-6.8-difluoro-7-hydroxycoumarin
COC	Cumulus-oocyte complexes
EV	Extracellular vesicles
EXO	Exosome
FA	Fatty acid
GSH	Glutathione
H2DCFDA	2′, 7′-dichlorodihydrofluorescein diacetates
HSL	Hormone-sensitive lipase
IACUC	Institutional Animal Care and Use Committee
IVC	In vitro culture
IVM	In vitro maturation
MGL	Monoacylglycerol Lipase
MMP	Mitochondrial membrane potential
NC	Negative control
NTA	Nanoparticle Tracking Analysis
PA	Parthenogenetic Activation
PC	Positive control
pFF	porcine follicular fluid
PFA/PBS	Paraformaldehyde/Phosphate buffered saline
PGC1α	Peroxisome proliferator-activated receptor-gamma coactivator-1alpha
Nrf1	Nuclear factor erythroid 2-related factor 1
PLIN3	Perilipin3
PZM	Porcine zygote media
ROS	Reactive oxygen species
TALP	Tyrode’s albumin lactate pyruvate
TCM199	Tissue culture media 199
TEM	Transmission electron microscope
TFAM	Mitochondrial transcription factor A

## References

[1] Yanez-Mo M., Siljander P.R., Andreu Z., Zavec A.B., Borras F.E., Buzas E.I., Buzas K., Casal E., Cappello F., Carvalho J. (2015). Biological properties of extracellular vesicles and their physiological functions. J. Extracell. Vesicles.

[2] Harris E.A., Stephens K.K., Winuthayanon W. (2020). Extracellular Vesicles and the Oviduct Function. Int. J. Mol. Sci..

[3] Thery C., Ostrowski M., Segura E. (2009). Membrane vesicles as conveyors of immune responses. Nat. Rev. Immunol..

[4] van Niel G., D’Angelo G., Raposo G. (2018). Shedding light on the cell biology of extracellular vesicles. Nat. Rev. Mol. Cell. Biol..

[5] Rodrigues T.A., Tuna K.M., Alli A.A., Tribulo P., Hansen P.J., Koh J., Paula-Lopes F.F. (2019). Follicular fluid exosomes act on the bovine oocyte to improve oocyte competence to support development and survival to heat shock. Reprod. Fertil. Dev..

[6] Gabrys J., Kij-Mitka B., Sawicki S., Kochan J., Nowak A., Lojko J., Karnas E., Bugno-Poniewierska M. (2022). Extracellular vesicles from follicular fluid may improve the nuclear maturation rate of in vitro matured mare oocytes. Theriogenology.

[7] Lee S.H., Saadeldin I.M. (2020). Exosomes as a Potential Tool for Supporting Canine Oocyte Development. Animals.

[8] Basuino L., Silveira C.F. (2016). Human follicular fluid and effects on reproduction. JBRA Assist. Reprod..

[9] Revelli A., Delle Piane L., Casano S., Molinari E., Massobrio M., Rinaudo P. (2009). Follicular fluid content and oocyte quality: From single biochemical markers to metabolomics. Reprod. Biol. Endocrinol..

[10] Brinca A.T., Ramalhinho A.C., Sousa A., Oliani A.H., Breitenfeld L., Passarinha L.A., Gallardo E. (2022). Follicular Fluid: A Powerful Tool for the Understanding and Diagnosis of Polycystic Ovary Syndrome. Biomedicines.

[11] Malyszka N., Pawlak P., Cieslak A., Szkudelska K., Lechniak D. (2023). Distinct dynamics of lipid accumulation by porcine cumulus cells during in vitro maturation with follicular fluid of low and high fatty acid contents. Theriogenology.

[12] Pawlak P., Warzych E., Cieslak A., Malyszka N., Maciejewska E., Madeja Z.E., Lechniak D. (2018). The consequences of porcine IVM medium supplementation with follicular fluid become reflected in embryo quality, yield and gene expression patterns. Sci. Rep..

[13] Reverchon M., Cornuau M., Rame C., Guerif F., Royere D., Dupont J. (2012). Chemerin inhibits IGF-1-induced progesterone and estradiol secretion in human granulosa cells. Hum. Reprod..

[14] Xie M., Li M., Zhou J., Ding X., Shao Y., Jing J., Liu Y., Yao B. (2017). Brain-derived neurotrophic factor promotes human granulosa-like tumor cell steroidogenesis and proliferation by activating the FSH receptor-mediated signaling pathway. Sci. Rep..

[15] Baumgarten S.C., Convissar S.M., Zamah A.M., Fierro M.A., Winston N.J., Scoccia B., Stocco C. (2015). FSH Regulates IGF-2 Expression in Human Granulosa Cells in an AKT-Dependent Manner. J. Clin. Endocrinol. Metab..

[16] Yuan C., Li Z., Zhao Y., Wang X., Chen L., Zhao Z., Cao M., Chen T., Iqbal T., Zhang B. (2021). Follicular fluid exosomes: Important modulator in proliferation and steroid synthesis of porcine granulosa cells. FASEB J..

[17] da Silveira J.C., Andrade G.M., Simas R.C., Martins-Junior H.A., Eberlin M.N., Smith L.C., Perecin F., Meirelles F.V. (2021). Lipid profile of extracellular vesicles and their relationship with bovine oocyte developmental competence: New players in intra follicular cell communication. Theriogenology.

[18] Wang W., Zhu N., Yan T., Shi Y.N., Chen J., Zhang C.J., Xie X.J., Liao D.F., Qin L. (2020). The crosstalk: Exosomes and lipid metabolism. Cell Commun. Signal..

[19] Mito T., Hoshi H. (2019). In Vitro Culture of Late Stage Pig Embryos in a Chemically Defined Medium, Porcine Blastocyst Medium (PBM). Methods Mol. Biol..

[20] Jiao X., Ding Z., Meng F., Zhang X., Wang Y., Chen F., Duan Z., Wu D., Zhang S., Miao Y. (2020). The toxic effects of Fluorene-9-bisphenol on porcine oocyte in vitro maturation. Environ. Toxicol..

[21] Li J., Wang R., Chen Q., Tian Y., Gao L., Lei A. (2022). Salidroside improves porcine oocyte maturation and subsequent embryonic development by promoting lipid metabolism. Theriogenology.

[22] Zhu T., Guan S., Lv D., Zhao M., Yan L., Shi L., Ji P., Zhang L., Liu G. (2021). Melatonin Modulates Lipid Metabolism in Porcine Cumulus-Oocyte Complex via Its Receptors. Front. Cell Dev. Biol..

[23] Lowe J.L., Bathgate R., Grupen C.G. (2019). Effect of carbohydrates on lipid metabolism during porcine oocyte IVM. Reprod. Fertil. Dev..

[24] Costermans N.G.J., Teerds K.J., Middelkoop A., Roelen B.A.J., Schoevers E.J., van Tol H.T.A., Laurenssen B., Koopmanschap R.E., Zhao Y., Blokland M. (2020). Consequences of negative energy balance on follicular development and oocyte quality in primiparous sowsdagger. Biol. Reprod..

[25] Kalluri R., LeBleu V.S. (2020). The biology, function, and biomedical applications of exosomes. Science.

[26] Jeppesen D.K., Fenix A.M., Franklin J.L., Higginbotham J.N., Zhang Q., Zimmerman L.J., Liebler D.C., Ping J., Liu Q., Evans R. (2019). Reassessment of Exosome Composition. Cell.

[27] Mashouri L., Yousefi H., Aref A.R., Ahadi A.M., Molaei F., Alahari S.K. (2019). Exosomes: Composition, biogenesis, and mechanisms in cancer metastasis and drug resistance. Mol. Cancer.

[28] Buratta S., Tancini B., Sagini K., Delo F., Chiaradia E., Urbanelli L., Emiliani C. (2020). Lysosomal Exocytosis, Exosome Release and Secretory Autophagy: The Autophagic- and Endo-Lysosomal Systems Go Extracellular. Int. J. Mol. Sci..

[29] Matsuno Y., Kanke T., Maruyama N., Fujii W., Naito K., Sugiura K. (2019). Characterization of mRNA profiles of the exosome-like vesicles in porcine follicular fluid. PLoS ONE.

[30] Yuan C., Chen X., Shen C., Chen L., Zhao Y., Wang X., Cao M., Zhao Z., Chen T., Zhang B. (2022). Follicular fluid exosomes regulate oxidative stress resistance, proliferation, and steroid synthesis in porcine theca cells. Theriogenology.

[31] Zhang W., Liu R., Chen Y., Wang M., Du J. (2022). Crosstalk between Oxidative Stress and Exosomes. Oxid. Med. Cell. Longev..

[32] Logozzi M., Mizzoni D., Di Raimo R., Fais S. (2020). Exosomes: A Source for New and Old Biomarkers in Cancer. Cancers.

[33] Xu R., Greening D.W., Zhu H.J., Takahashi N., Simpson R.J. (2016). Extracellular vesicle isolation and characterization: Toward clinical application. J. Clin. Investig..

[34] Lobb R.J., Becker M., Wen S.W., Wong C.S., Wiegmans A.P., Leimgruber A., Moller A. (2015). Optimized exosome isolation protocol for cell culture supernatant and human plasma. J. Extracell. Vesicles.

[35] Saeed-Zidane M., Linden L., Salilew-Wondim D., Held E., Neuhoff C., Tholen E., Hoelker M., Schellander K., Tesfaye D. (2017). Cellular and exosome mediated molecular defense mechanism in bovine granulosa cells exposed to oxidative stress. PLoS ONE.

[36] MacPherson R.E., Ramos S.V., Vandenboom R., Roy B.D., Peters S.J. (2013). Skeletal muscle PLIN proteins, ATGL and CGI-58, interactions at rest and following stimulated contraction. Am. J. Physiol. Regul. Integr. Comp. Physiol..

[37] Skryabin G.O., Komelkov A.V., Savelyeva E.E., Tchevkina E.M. (2020). Lipid Rafts in Exosome Biogenesis. Biochemistry.

[38] Kim E.H., Ridlo M.R., Lee B.C., Kim G.A. (2021). Crosstalk between Peroxisomal Activities and Nrf2 Signaling in Porcine Embryos. Antioxidants.

[39] Kim E.H., Ridlo M.R., Lee B.C., Kim G.A. (2020). Melatonin-Nrf2 Signaling Activates Peroxisomal Activities in Porcine Cumulus Cell-Oocyte Complexes. Antioxidants.

[40] Kim E.H., Kim G.A., Taweechaipaisankul A., Ridlo M.R., Lee S.H., Ra K., Ahn C., Lee B.C. (2020). Phytanic acid-derived peroxisomal lipid metabolism in porcine oocytes. Theriogenology.

[41] Kim E.H., Kim G.A., Taweechaipaisankul A., Lee S.H., Qasim M., Ahn C., Lee B.C. (2019). Melatonin enhances porcine embryo development via the Nrf2/ARE signaling pathway. J. Mol. Endocrinol..

[42] Benador I.Y., Veliova M., Liesa M., Shirihai O.S. (2019). Mitochondria Bound to Lipid Droplets: Where Mitochondrial Dynamics Regulate Lipid Storage and Utilization. Cell. Metab..

[43] Park J.E., Lee S.H., Hwangbo Y., Park C.K. (2021). Porcine follicular fluid derived from > 8 mm sized follicles improves oocyte maturation and embryo development during in vitro maturation of pigs. Zygote.

[44] Jin J.X., Lee S., Setyawan E.M.N., Taweechaipaisankul A., Kim G.A., Han H.J., Ahn C., Lee B.C. (2018). A potential role of knockout serum replacement as a porcine follicular fluid substitute for in vitro maturation: Lipid metabolism approach. J. Cell. Physiol..

[45] Srivastava A., Srivastava P., Mathur S., Abbas S., Rai N., Tiwari S., Tiwari M., Sharma L. (2022). Lipid Metabolism and Mitochondria: Cross Talk in Cancer. Curr. Drug Targets.

[46] Brownsey R.W., Boone A.N., Elliott J.E., Kulpa J.E., Lee W.M. (2006). Regulation of acetyl-CoA carboxylase. Biochem. Soc. Trans..

[47] Chen L., Duan Y., Wei H., Ning H., Bi C., Zhao Y., Qin Y., Li Y. (2019). Acetyl-CoA carboxylase (ACC) as a therapeutic target for metabolic syndrome and recent developments in ACC1/2 inhibitors. Expert. Opin. Investig. Drugs.

[48] Grabner G.F., Xie H., Schweiger M., Zechner R. (2021). Lipolysis: Cellular mechanisms for lipid mobilization from fat stores. Nat. Metab..

[49] Duncan R.E., Ahmadian M., Jaworski K., Sarkadi-Nagy E., Sul H.S. (2007). Regulation of lipolysis in adipocytes. Annu. Rev. Nutr..

[50] Bai L., Gong J., Guo Y., Li Y., Huang H., Liu X. (2022). Construction of a ceRNA network in polycystic ovary syndrome (PCOS) driven by exosomal lncRNA. Front. Genet..

[51] Saadeldin I.M., Kim S.J., Choi Y.B., Lee B.C. (2014). Improvement of cloned embryos development by co-culturing with parthenotes: A possible role of exosomes/microvesicles for embryos paracrine communication. Cell. Reprogram.

[52] Kim E.H., Taweechaipaisankul A., Ridlo M.R., Kim G.A., Lee B.C. (2020). Effect of Klotho protein during porcine oocyte maturation via Wnt signaling. Aging.

[53] Jin J.X., Lee S., Taweechaipaisankul A., Kim G.A., Lee B.C. (2017). Melatonin regulates lipid metabolism in porcine oocytes. J. Pineal Res..

[54] Ducolomb Y., Gonzalez-Marquez H., Fierro R., Jimenez I., Casas E., Flores D., Bonilla E., Salazar Z., Betancourt M. (2013). Effect of porcine follicular fluid proteins and peptides on oocyte maturation and their subsequent effect on in vitro fertilization. Theriogenology.

[55] de Andrade Melo-Sterza F., Poehland R. (2021). Lipid Metabolism in Bovine Oocytes and Early Embryos under In Vivo, In Vitro, and Stress Conditions. Int. J. Mol. Sci..

[56] Momozawa K. (2020). Usefulness of modified Medium RD as a chemically defined medium for in vitro maturation of bovine oocytes. Reprod. Med. Biol..

